# Supplementary material for: Hydrogen peroxide signaling via its transformation to a stereospecific alkyl hydroperoxide that escapes reductive inactivation
Source: Nat Commun. 2021 Nov 16;12:6626. doi: 10.1038/s41467-021-26991-5 (PMC8595612; doi:10.1038/s41467-021-26991-5)
Supplement: Supplementary file 1 — Supplementary Information [file 41467_2021_26991_MOESM1_ESM.pdf]

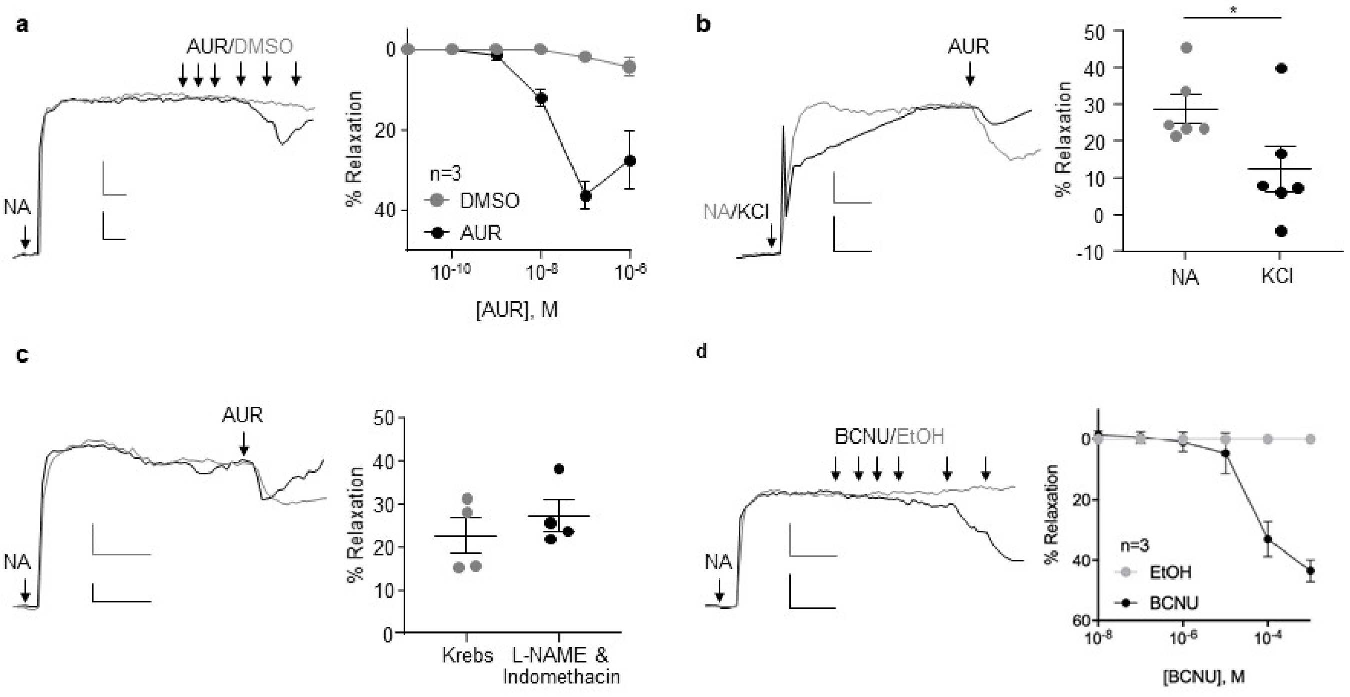

Supplementary Fig. 1. Effect of AUR and BCNU on arterial relaxation. a) Concentration-dependent relaxation responses to auranofin (AUR) compared with vehicle (DMSO) control in mesenteric arteries isolated from naïve C57BL6/J mice and pre-constricted with noradrenaline (NA) (*n*=3). **b)** Comparison of relaxation responses to 300 nM AUR in mesenteric arteries isolated from naïve C57BL6/J mice and pre-constricted with NA- or KCl (120 mM) (*n*=6; *p* = 0.0411). **c)** Relaxation responses to 300 nM AUR of mesenteric arteries isolated from naïve C57BL6/J mice and pre-constricted with NA in Krebs buffer ± 100 µM L-N$^{\omega}$-Nitro arginine methyl ester (L-NAME) and 10 µM indomethacin (*n*=4). **d** Concentration-dependent relaxation responses to [1,3-*bis*(2-chloroethyl)-1-nitrosourea] (BCNU) or vehicle (ethanol, EtOH) in mesenteric arteries isolated from naïve C57BL6/J mice and pre-constricted with NA (*n*=3). Representative traces and respective dot blots shown. Summary data is shown as mean ± SEM, with each individual data point referring to an independent experiment. Statistical analysis was performed using two-tailed Mann-Whitney tests, *$p \le 0.05$.

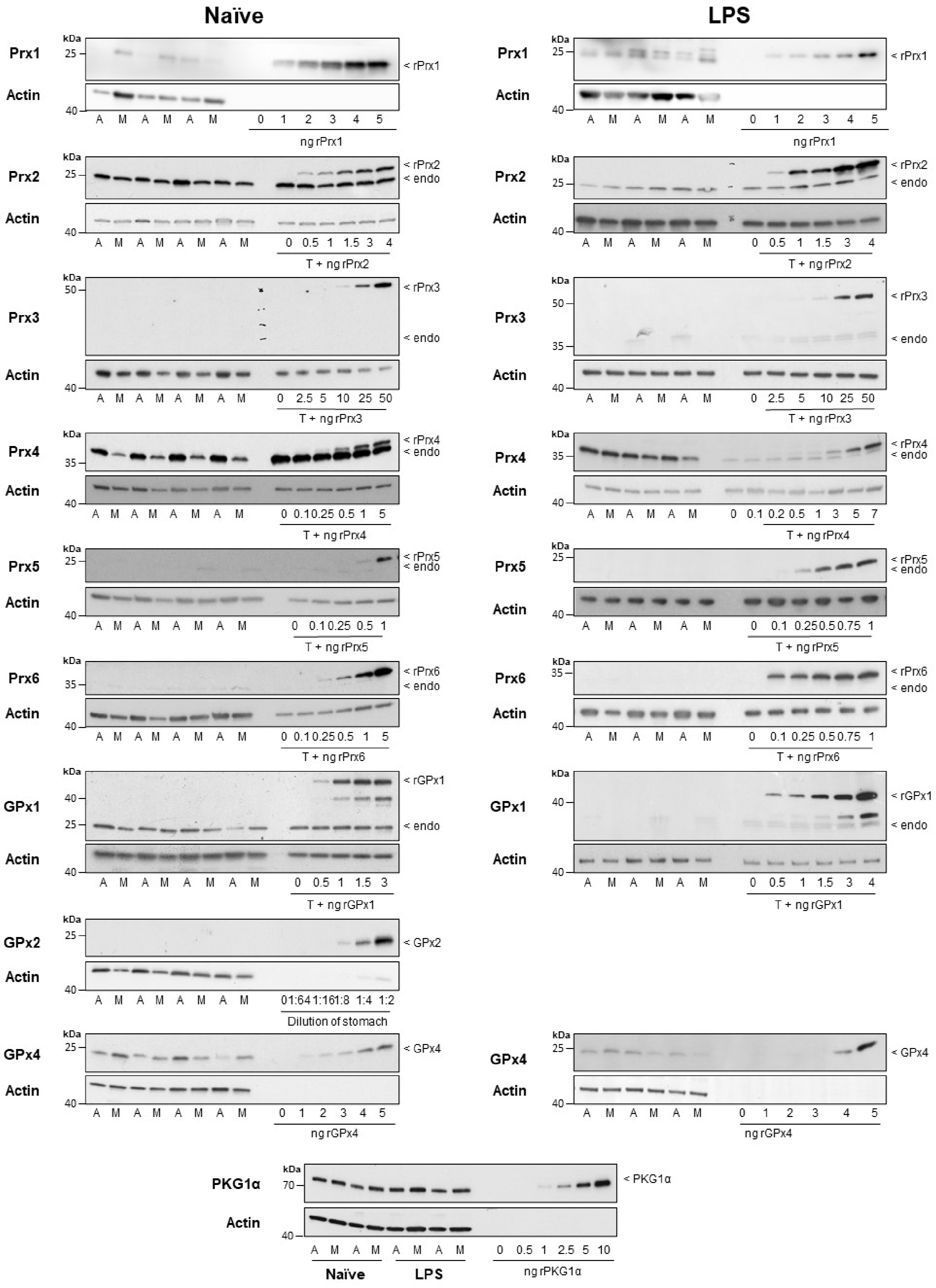

Supplementary Fig. 2. Expression of peroxiredoxins (Prx), glutathione peroxidases (GPx) and PKG1α in arteries from naïve and LPS-treated mice. Representative immunoblots used to quantify the

expression of Prx1, Prx2, Prx3, Prx4, Prx5, Prx6, GPx1, GPx2, GPx4 and PKG1α in abdominal aorta (A) and mesenteric arteries (M) from naïve and LPS-treated mice. Arteries from individual mice were pooled separately (4 aortas and 6 mesenteric arterial beds in each pool; with each pool considered an independent replicate), except for Prx1 expression that was assessed using arteries from three different naïve and LPS-treated animals. Endogenous (endo) protein expression was determined against a standard curve of each respective recombinant (r) protein, run alone or in a matrix of thoracic aorta homogenate (T), except for GPx2 which was measured only in naïve arteries using a surrogate standard curve of decreasing dilutions of mouse stomach homogenate, a tissue known to express GPx2. Expression of Prx2, GPx4 and PKG1α were calculated using 7 μg of total arterial homogenate, Prx4 in 10 μg and all other isoforms in 15-25 μg. n=4 independent arterial pools (naïve) and n=3 independent arterial pools (LPS-treated). All replicates are shown in the Figure.

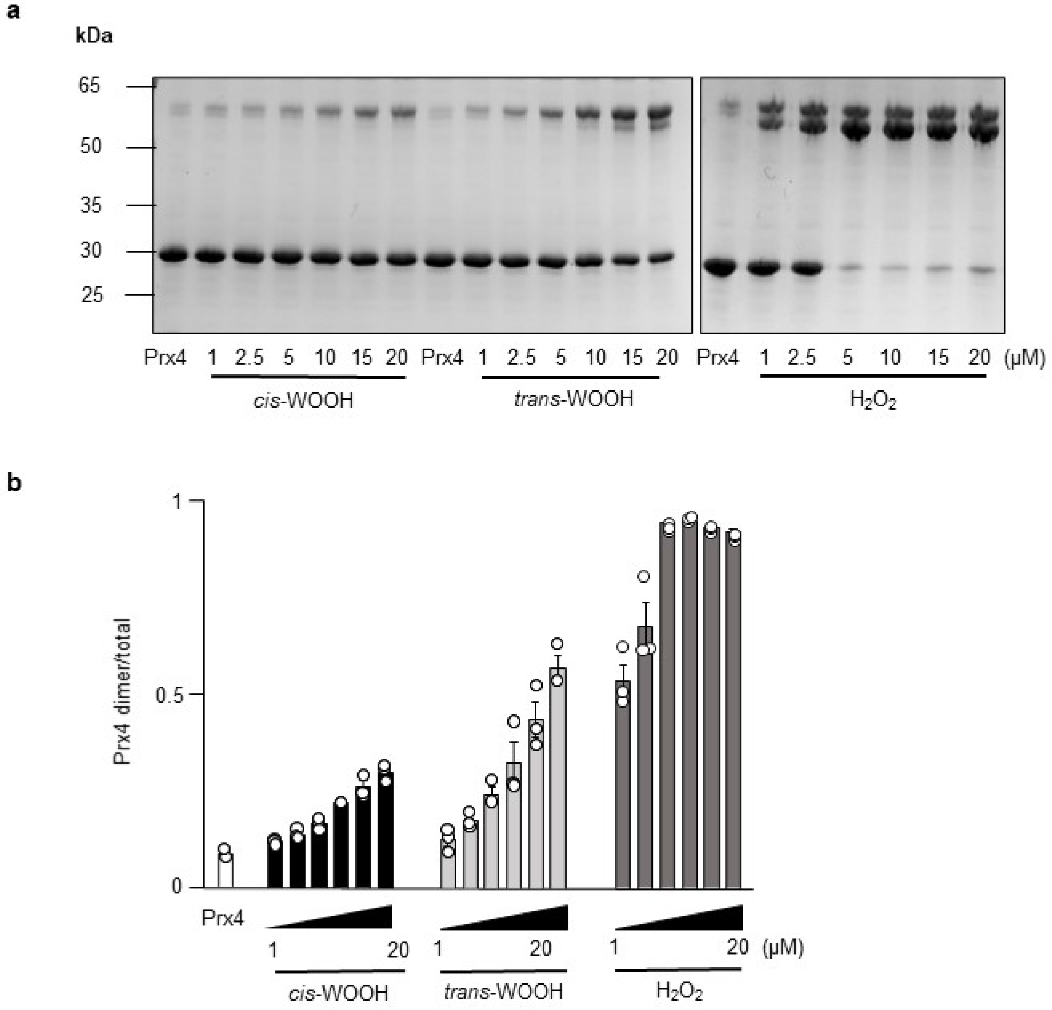

Supplementary Fig. 3. *cis*- and *trans*-WOOH are inefficiently reduced by His-tagged Prx4. a) Immediately after pre-reduction, His-tagged Prx4 (20 µM) was incubated with 1-20 µM *cis*-WOOH or *trans*-WOOH in 50 mM sodium phosphate buffer (pH 7.4, 5 °C). After 10 s, 50 mM NEM was added before subjecting the samples to non-reducing 4-12% SDS-PAGE and Silver staining the gels. H_2O_2 (1-20 µM) was used as a positive control. Data shown are representative of three separate experiments. **b**) Quantification of silver-stained Prx4 dimer and monomer bands in **a**) with results expressed as ratio of Prx4 dimer-to-monomer. After 10 s, ~25% Prx4 dimer was formed by equimolar *cis*-WOOH, corresponding to ~0.5 µM Prx4 dimer s^{-1}. After 10 s, ~60% Prx4 dimer was formed by equimolar *trans*-WOOH, corresponding to ~1.2 µM Prx4 dimer s^{-1}. From the rate equation (v = k x [Prx4] x [WOOH]) a second order rate constant for the reaction of *cis*- and *trans*-WOOH and Prx4 was estimated as ~1,200 $M^{-1}s^{-1}$ and ~3000 $M^{-1}s^{-1}$, respectively. Data in (**b**) shows the mean ± SEM, n=3 independent experiments.

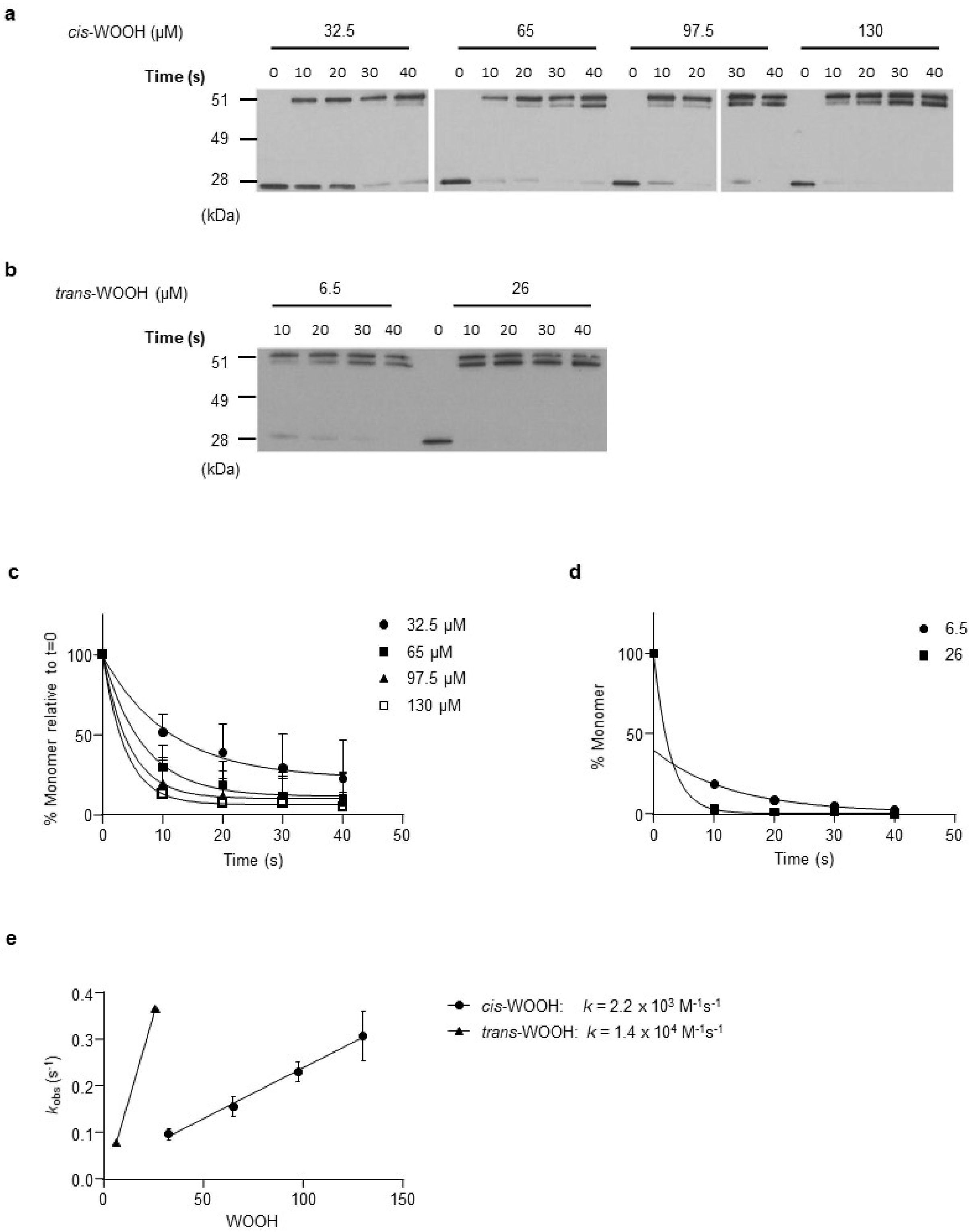

Supplementary Fig. 4. Estimation of the rate constant for the reaction of *cis*- and *trans*-WOOH with untagged wild-type Prx2 by redox blotting. a) Reaction of pre-reduced Prx2 (5 µM) with *cis*-WOOH (32.5-130 µM) at 5 °C. After incubation, NEM (20 mM) was added, and the samples subjected to non-reducing SDS-PAGE followed by Western blotting using anti-Prx2 antibody. **b**) Reaction of pre-reduced Prx2 (5 µM) with *trans*-WOOH (6.5-26 µM) at 5 °C. After incubation, NEM (20 mM) was added, and the samples subjected to non-reducing SDS-PAGE followed by Western blotting using anti-Prx2 antibody. **c, d**) Time course of monomer disappearance in the presence of increasing concentrations of *cis*-WOOH (**c**) or *trans*-WOOH (**d**). **e**) Determination of second-order rate constant for the reaction of Prx2 with *cis*-WOOH ($2.2 \times 10^3\ M^{-1}s^{-1}$ at 5 °C) and *trans*-WOOH ($1.4 \times 10^4\ M^{-1}s^{-1}$) at 5 °C. Blots (**a**, **b**) and decay plots (**c**, **d**) are representative of five separate experiments (*cis*-WOOH) or one experiment (*trans*-WOOH). Data in (**e**) shows the mean ± SEM of five independent experiments (*cis*-WOOH) and absolute values from one experiment (*trans* WOOH).

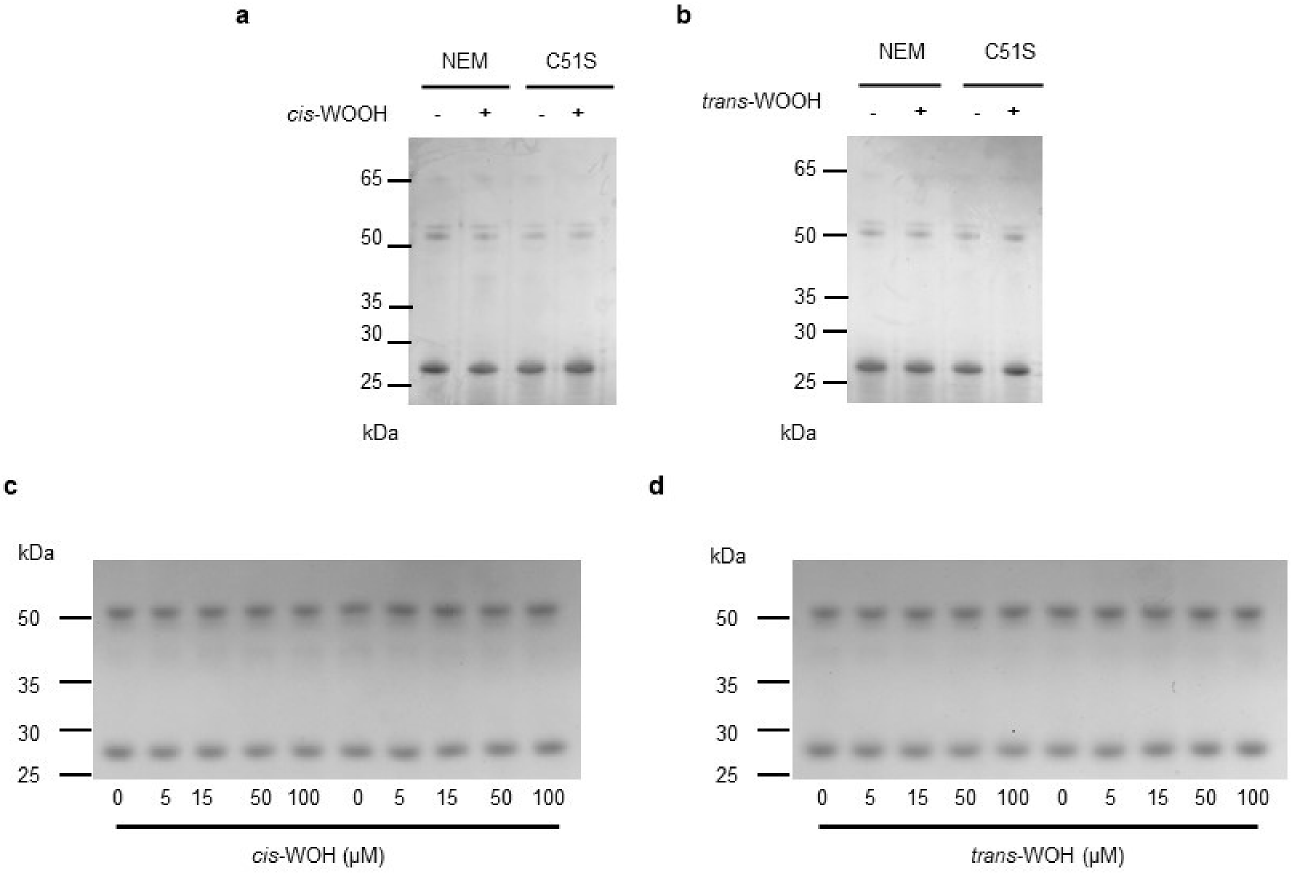

Supplementary Fig. 5. Dimerization of His-tagged Prx2 depends on its peroxidatic thiol residue and a hydroperoxide. Pre-alkylated His-tagged Prx2 (NEM) or His-tagged Prx2 C51S (5 µM) was incubated at 25 °C with 40 µM *cis*-WOOH (**a**) or *trans*-WOOH (b) for 60 s before addition of *N*-ethylmaleimide (NEM; 30 mM) and non-reducing 4-12% SDS-PAGE and silver staining of gels. Results shown are representative of two separate experiments. His-tagged Prx2 (5 µM) was incubated with the indicated concentration of *cis*- (**c**) or *trans*-WOH (**d**) for 5 min at 25 °C. After incubation, NEM (30 mM) was added, and the samples subjected to non-reducing 4-12% SDS-PAGE followed by overnight Colloidal Coomassie staining of gels. For **c** and **d**) results from two separate experiments are shown

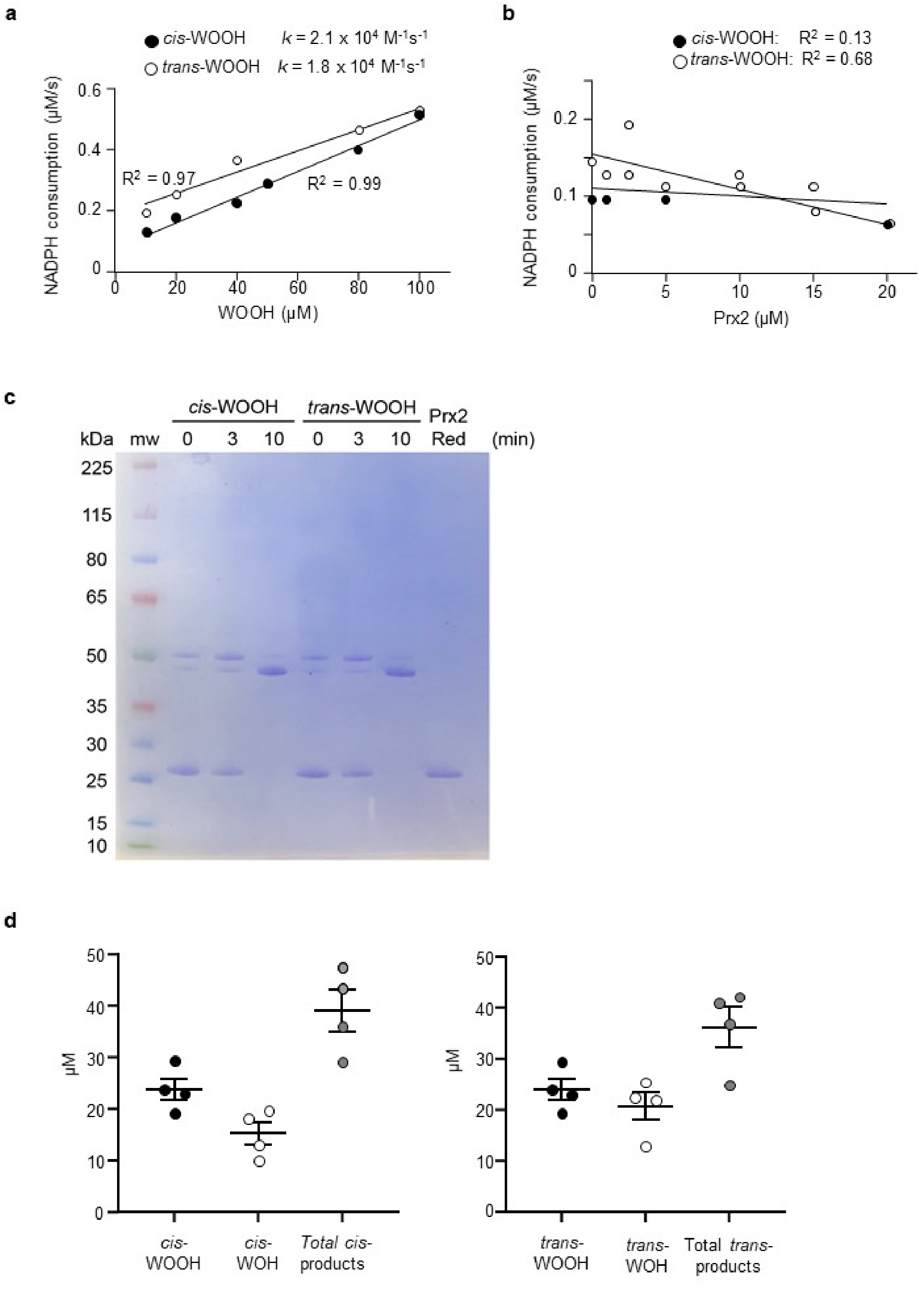

Supplementary Fig. 6. Thioredoxin reductase competition experiments to validate the rate constant for the reaction of Prx2 with *cis*- and *trans*-WOOH determined by redox blotting. **a**) Determination of the rate constant between the reaction of rat thioredoxin reductase (TrxR, 0.2 µM) and *cis*-WOOH and *trans*-WOOH following NADPH consumption at 340 nm at 25 °C. Rates of reaction between *cis*- and *trans*-WOOH and TrxR were determined using the initial rate approach (2.1 x 10^4 and 1.8 x 10^4 $M^{-1}s^{-1}$ for *cis*- and *trans*-WOOH, respectively). **b)** Competition assay between TrxR (0.2 µM) and His-tagged Prx2 (≤20 µM) for reaction with *cis*-WOOH (10 µM) and *trans*-WOOH (10 µM). NADPH consumption was followed spectrophotometrically at 340 nm at 25 °C, with the rates of reaction between *cis*- and *trans*-WOOH and TrxR in the

presence of Prx2 determined using the initial rate approach. **c**) To ensure Prx2 remained reduced/active during experiments with TrxR, pre-reduced His-tagged Prx2 (10 μM) was incubated at 25 °C for 3 min in HEPES buffer containing NADPH (200 μM) and TrxR (0.2 μM), before *cis*- or *trans*-WOOH (10 μM) was added and the reaction incubated for a further 7 min. Aliquots of the reaction mixture were removed 0, 3 and 10 min after the start of the reaction, alkylated with NEM and subjected to non-reducing 4-12% SDS-PAGE and Coomassie staining. (**d**) To demonstrate TrxR dependent reduction of *cis*- and *trans*-WOOH, TrxR (0.2 μM), NADPH (200 μM) and *cis*- or *trans*-WOOH (50 μM) were incubated at 25 °C in HEPES buffer for 10 min and then analyzed by LC-MS/MS for WOOH and WOH. (**c**) shows a representative blot. (**d**) shows total products (*cis*-WOOH and *cis*-WOH, *trans*-WOOH and *trans*-WOH) as mean ± SEM, n= 4 independent enzyme assays.

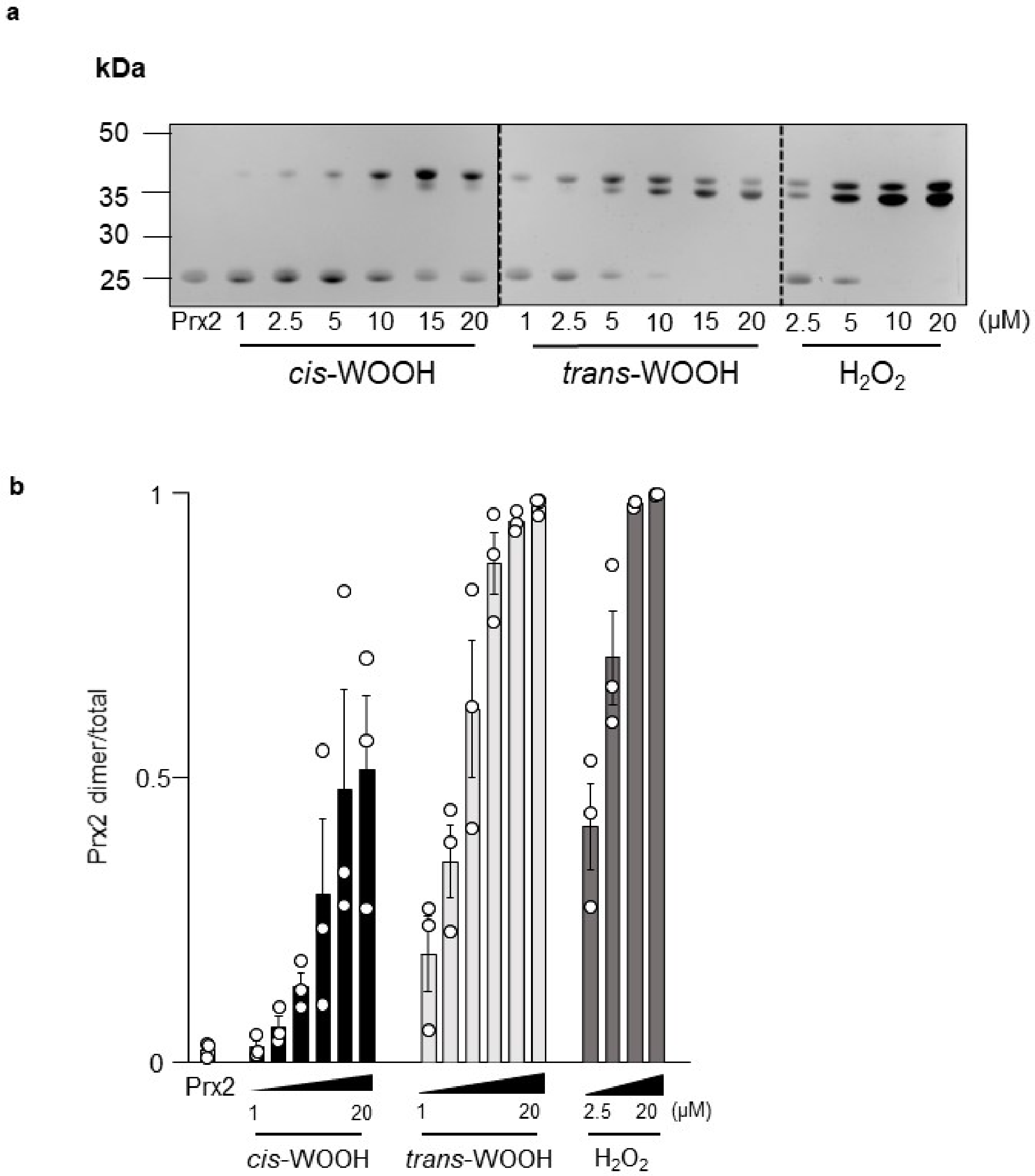

Supplementary Fig. 7. **Dimerization of untagged Prx2 by *cis*- and *trans*-WOOH**. **a**) Immediately after pre-reduction, untagged Prx2 (20 µM) was incubated with 1-20 µM *cis*-WOOH or *trans*-WOOH in 50 mM sodium phosphate buffer (pH 7.4, 5 °C). After 10 s, 50 mM NEM was added before subjecting the samples to non-reducing 4-12% SDS-PAGE and Silver staining the gels. H_2O_2 (2.5-20 µM) was used as a positive control. Data shown are representative of three separate experiments. **b**) Quantification of silver-stained Prx2 dimer and monomer bands in a) with results expressed as ratio of Prx2 dimer-to-monomer Data in (**b**) shows the mean ± SEM, n=3 independent experiments.

a

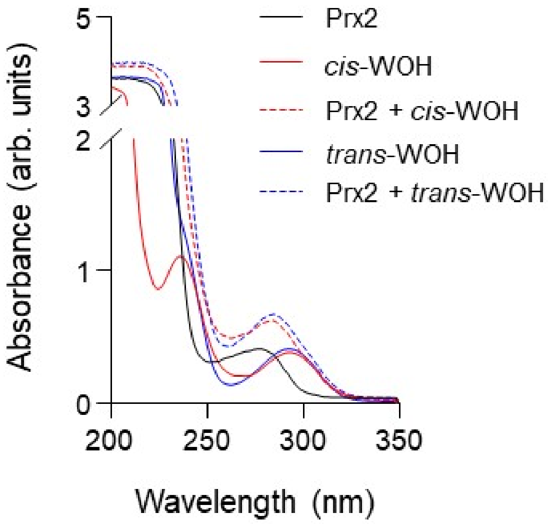

b

***cis*-WOH (µM)**	**Δ (nm)**
100	13
200	9
400	7
***trans*-WOH (µM)**	**Δ (nm)**
100	11
200	7
400	6

Supplementary Fig. 8. Changes in absorbance spectra of *cis*- or *trans*-WOH in the presence of untagged Prx2. **a**) Immediately after pre-reduction, untagged Prx2 (20 µM) was incubated with 200 µM *cis*- or *trans*-WOH in 50 mM sodium phosphate buffer (pH 7.4, 25 °C) for 10 s before spectral scanning from 200 to 350 nm. **b**) Representation of data from **a**) as delta of maximum absorption wavelength for both alcohols (100, 200 and 400 µM) in the absence and presence of Prx2. The values shown are mean of two independent experiments.

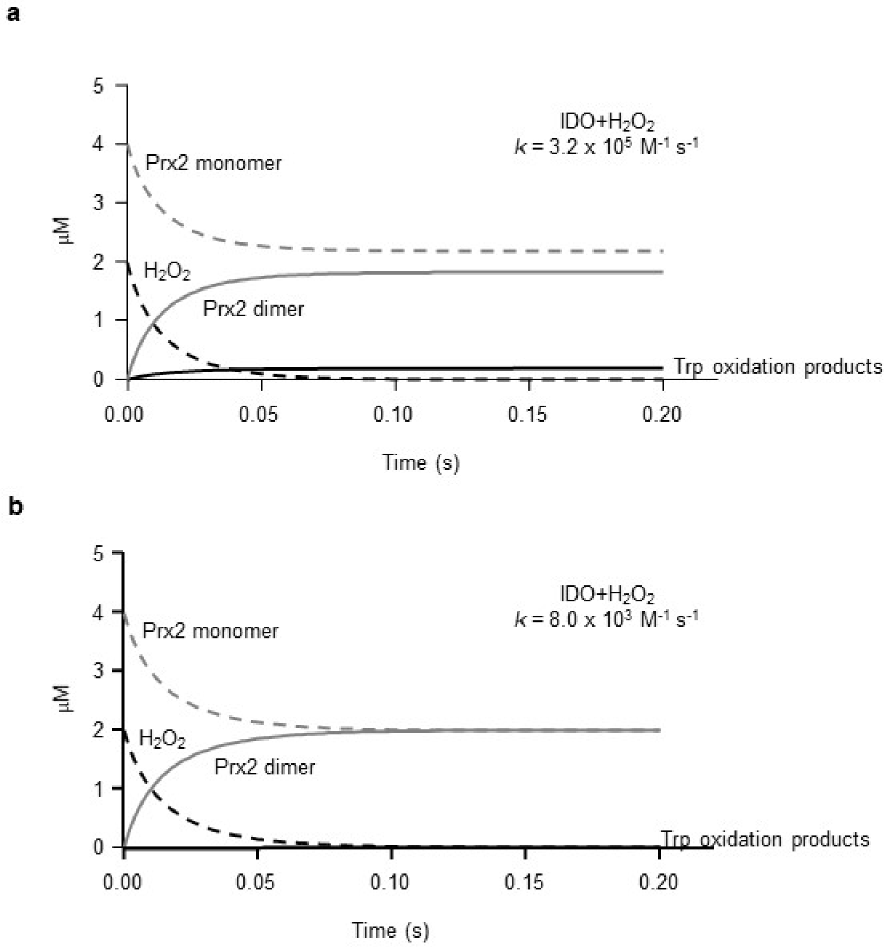

Supplementary Fig. 9. *In silico* simulation of formation of Trp oxidation products assuming different rate constants for the reaction of IDO1 with H_2O_2. Formation of H_2O_2-derived Trp oxidation products were simulated using the following conditions: 4 μM Prx2, 2 μM H_2O_2, 100 μM L-Trp and 20 μM IDO1. The rate of H_2O_2 reaction with Prx2 was set at $k = 2 \times 10^7$ $M^{-1}s^{-1}$ and the rate of reaction for IDO1+H_2O_2 was set at either 3.2 x 10^5 $M^{-1}s^{-1}$ (**a**) or 8.0 x 10^3 $M^{-1}s^{-1}$ (**b**). All simulations were done using Gepasi biochemical simulation software.

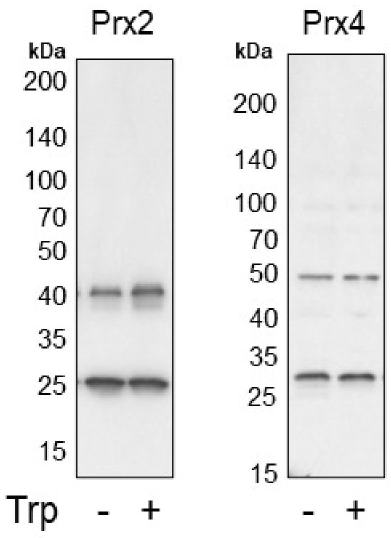

Supplementary Fig. 10. Uncropped immunoblots for Prx2 and Prx4 from Fig. 7. Representative uncropped immunoblots for Prx2 and Prx4 in mesenteric arteries from LPS-injected mice ± 8 mM L-Trp *iv* injection for 1 min. Blot is representative of one of three independent replicates.
