# Supplementary material for: Hydrogen peroxide signaling via its transformation to a stereospecific alkyl hydroperoxide that escapes reductive inactivation
Source: Nat Commun. 2021 Nov 16;12:6626. doi: 10.1038/s41467-021-26991-5 (PMC8595612; doi:10.1038/s41467-021-26991-5)
Supplement: Supplementary file 3 — Reporting Summary [file 41467_2021_26991_MOESM3_ESM.pdf]

nature portfolio

Corresponding author(s): Anita Ayer and Roland Stcoker

Last updated by author(s): Sep 29, 2021

Reporting Summary

Nature Portfolio wishes to improve the reproducibility of the work that we publish. This form provides structure for consistency and transparency in reporting. For further information on Nature Portfolio policies, see our Editorial Policies and the Editorial Policy Checklist.

Statistics

For all statistical analyses, confirm that the following items are present in the figure legend, table legend, main text, or Methods section.

n/a	Confirmed	
☐	☒	The exact sample size (n) for each experimental group/condition, given as a discrete number and unit of measurement
☐	☒	A statement on whether measurements were taken from distinct samples or whether the same sample was measured repeatedly
☐	☒	The statistical test(s) used AND whether they are one- or two-sided<br>*Only common tests should be described solely by name; describe more complex techniques in the Methods section.*
☒	☐	A description of all covariates tested
☒	☐	A description of any assumptions or corrections, such as tests of normality and adjustment for multiple comparisons
☐	☒	A full description of the statistical parameters including central tendency (e.g. means) or other basic estimates (e.g. regression coefficient) AND variation (e.g. standard deviation) or associated estimates of uncertainty (e.g. confidence intervals)
☐	☒	For null hypothesis testing, the test statistic (e.g. F, t, r) with confidence intervals, effect sizes, degrees of freedom and P value noted<br>*Give P values as exact values whenever suitable.*
☒	☐	For Bayesian analysis, information on the choice of priors and Markov chain Monte Carlo settings
☒	☐	For hierarchical and complex designs, identification of the appropriate level for tests and full reporting of outcomes
☒	☐	Estimates of effect sizes (e.g. Cohen's d, Pearson's r), indicating how they were calculated

Our web collection on statistics for biologists contains articles on many of the points above.

Software and code

Policy information about availability of computer code

Data collection	LC-MS/MS data was collected using Agilent Technologies MassHunter Workstation software (version B.06.00 Build 6.0.6025.0; and analysis was performed using Mass Hunter Qualitative Analysis version B.06.00 Build 6.0.633.10 Service pack 1), or Thermo Scientific Xcalibur software (Version 4.4.16.14). Native mass spectrometry data was collected using Thermo Fisher XCalibur software (v2.2 SP1.48) and analyzed using MATLAB software. (vR2019b) Spectrophotometric data was collected using Agilent Technologies Cary WinUV software (v3.0), Shimadzu UV Probe software (v2.33), Molecular Devices' SoftMax Pro Microplate Data Acquisition and Analysis software (v6). Arterial relaxation data was collected using ADI Lab Chart 8 software. (v8.1.17) Western blot or protein staining (Coomassie or silver) densitometry data was collected using Image Studio Lite (LI-COR biosciences, v5.2.5) or Image J (v1.51). Chemical structures were drawn using ChemDraw Professional 17.0. Kinetic simulations were conducted using GEPASI v3.30 software.
Data analysis	Statistical analyses were carried out using GraphPad Prism (v8.4.3) or Microsoft Excel 365 (version 2108, Build 14326.20404)

For manuscripts utilizing custom algorithms or software that are central to the research but not yet described in published literature, software must be made available to editors and reviewers. We strongly encourage code deposition in a community repository (e.g. GitHub). See the Nature Portfolio guidelines for submitting code & software for further information.

Data

Policy information about availability of data

All manuscripts must include a data availability statement. This statement should provide the following information, where applicable:

- Accession codes, unique identifiers, or web links for publicly available datasets
- A description of any restrictions on data availability
- For clinical datasets or third party data, please ensure that the statement adheres to our policy

All data generated or analyzed during this study are included in this published article (and its supplementary information files). Information pertaining to respective recombinant and endogenous proteins was obtained from Uniprot. Accession numbers are follows: Prx1 (P35700), Prx2 (Q61171), Prx2 (P20108), Prx4 (O08807), Prx5 (P99029), Prx6 (O08709), GPx1 (P11352) and GPx4 (O70325).

Field-specific reporting

Please select the one below that is the best fit for your research. If you are not sure, read the appropriate sections before making your selection.

☒ Life sciences ☐ Behavioural & social sciences ☐ Ecological, evolutionary & environmental sciences

For a reference copy of the document with all sections, see nature.com/documents/nr-reporting-summary-flat.pdf

Life sciences study design

All studies must disclose on these points even when the disclosure is negative.

Sample size	Sample sizes were not determined statistically. For physiological experiments (in vivo and ex vivo), enzymatic and analytical experiments samples sizes were chosen based on Nature 566, 584-552 (2019). Independent experiments refers to biologically independent experiments.
Data exclusions	In arterial relaxant studies arteries were validated by their ability to relax ≥80 to a nitric oxide donor at the end of the experiment. Any arteries which failed to fulfill this pre-established criteria were excluded from analysis.
Replication	Reproducibility of the experimental findings was verified by using positive and negative controls to test intra- and inter-day variation. If a positive control did not provide a signal or result in a pre-specified range the assay on the day was deemed not reproducible and test samples were thus not tested or, if tested, data not analyzed nor included in final results. Experiments would then be repeated to ensure that positive and negative (where required) controls provided data within reasonable pre-specified range (usually 20% inter-day variation and 10% intraday variation). Experiments were performed independently a minimum of three times, unless otherwise indicated in the manuscript. Data from all attempts is shown in the manuscript and provided in Source Data.
Randomization	In all animal studies, all drug and/or inhibitor treatment groups were randomized between vessels. For all other experiments (biochemical and enzyme assays), randomization was not carried out, as it was not feasible.
Blinding	Blinding was not possible as individual experiments and analyses were carried out by single operator.

Reporting for specific materials, systems and methods

We require information from authors about some types of materials, experimental systems and methods used in many studies. Here, indicate whether each material, system or method listed is relevant to your study. If you are not sure if a list item applies to your research, read the appropriate section before selecting a response.

Materials & experimental systems

n/a	Involved in the study	
☐	☒	Antibodies
☐	☒	Eukaryotic cell lines
☒	☐	Palaeontology and archaeology
☐	☒	Animals and other organisms
☒	☐	Human research participants
☒	☐	Clinical data
☒	☐	Dual use research of concern

Methods

n/a	Involved in the study	
☒	☐	ChIP-seq
☒	☐	Flow cytometry
☒	☐	MRI-based neuroimaging

Antibodies

Antibodies used	Primary antibodies: Prx1 (1:1,000 dilution, vol/vol, AdipoGen, YIF-LF-MA0214), Prx2 (1:2,000 dilution, vol/vol, Sigma, R8656), Prx3 (1:500 dilution, vol/vol, Abfrontier, LF-PA0030), Prx4 (1:2,000 dilution, vol/vol, Abcam, ab184167), Prx5 (1:2,000 dilution, vol/vol, Abcam, ab180587), Prx6 (1:2,000 dilution, vol/vol, Abcam, ab59543), GPx1 (1:2,000 dilution, vol/vol, Abcam ab62204), GPx2 (1:500

	dilution, vol/vol, Sigma Aldrich SAB2700206, GPx4 (1:2,000 dilution, vol/vol, Abcam ab125066), PKG1α (1:1,000 dilution, vol/vol, ENZO Life Sciences, ADI-KAP-PK005), Prx-SO2/3 (1:1000 dilution, vol/vol, Abcam ab16830) antibody or mouse polyclonal anti-actin (1:5,000 dilution, vol/vol, MP Biomedicals, 8691001) antibody. Secondary antibodies: Polyclonal goat anti-rabbit horseradish peroxidase-conjugated IgG (1:5,000 dilution, vol/vol, Dako P0446) for Prx, GPx and PKG1α, or polyclonal goat anti-mouse horseradish peroxidase-conjugated IgG (1:5,000 dilution, vol/vol, Dako P0447) for actin.
Validation	Primary antibodies to detect proteins of interest were validated in murine tissue homogenates with known expression of each target protein and also by using purified recombinant proteins as described in-text (see Methods and supporting Figures).

Eukaryotic cell lines

Policy information about cell lines

Cell line source(s)	FreeStyle™ 293-F cells (Gibco™ R79007)
Authentication	Cell lines were not authenticated
Mycoplasma contamination	Cell lines tested negative for mycoplasma contamination
Commonly misidentified lines (See ICLAC register)	Not applicable

Animals and other organisms

Policy information about studies involving animals; ARRIVE guidelines recommended for reporting animal research

Laboratory animals	We used 8-12 weeks-old male mice that were either wild type C57BL/6J or PKG1α C42S mutant mice on C57BL/6N background. Animals were housed in rooms with a 12 h light/dark cycle corresponding to sunrise and sunset of approximately 6 am and 6 pm, respectively. The room temperature was maintained between 20-26 °C with a humidity set between 40-70%.
Wild animals	No wild animals were used
Field-collected samples	No field samples were collected
Ethics oversight	Animal Ethics Committees of the Garvan Institute of Medical Research/St Vincent's Hospital (Protocols 15_34 and 18_30) and Sydney Local Health District (SLHD AWC 2020-2026 and SLHD AWC 2020-027.

Note that full information on the approval of the study protocol must also be provided in the manuscript.
